# Higher spatial resolution diffusion-weighted imaging improves characterization of white matter disconnection in Alzheimer’s disease

**DOI:** 10.1162/IMAG.a.1078

**Published:** 2026-01-07

**Authors:** Jenna L. Merenstein, Allen W. Song, Kim G. Johnson, Heather E. Whitson, David J. Madden

**Affiliations:** Department of Psychology, University of Utah, Salt Lake City, UT, United States; Brain Imaging and Analysis Center, Duke University Medical Center, Durham, NC, United States; Department of Psychiatry and Behavioral Sciences, Duke University Medical Center, Durham, NC, United States; Center for the Study of Aging and Human Development, Duke University Medical Center, Durham, NC, United States; Durham VA Geriatrics Research Education and Clinical Center, Durham, NC, United States

**Keywords:** magnetic resonance imaging, graph theoretical analyses, structural connectivity, system segregation, multiplexed sensitivity encoding, neurodegeneration

## Abstract

In addition to the accumulation of neuropathologies (i.e., amyloid beta, neurofibrillary tangles), Alzheimer’s disease (AD) is associated with the degradation of white matter (WM) structural pathways that connect distributed brain regions. However, previous studies of AD-related decreases in WM structural connectivity have primarily used standard spatial resolution diffusion-weighted imaging (DWI) acquisitions, which cannot adequately resolve WM connections in fine-grained regions with crossing fibers or high curvature. To better assess more subtle WM tissue degradation in AD, this DWI study compared measures of structural connectivity derived from a high-resolution multi-shot multiplexed sensitivity encoding (MUSE) acquisition (1 mm isotropic voxels; 1 µl volume) and a standard resolution acquisition (1.5 mm isotropic voxels; 3.375 µl volume), using a sample of 24 participants with AD and 24 demographically matched healthy controls. Graph theoretical measures of within-network connectivity, between-network connectivity, and system segregation were obtained from a standard network partition. As expected, results indicated larger AD-related decreases in between-network than within-network connectivity, leading to increased network segregation for both DWI acquisitions. However, the high-resolution MUSE DWI acquisition achieved higher sensitivity and specificity to AD-related differences in structural connectivity than the standard resolution DWI protocol, consistent with prior findings from rodents and healthy adults across the lifespan. Together, these findings suggest that this clinically feasible, high-resolution MUSE DWI methodology may detect more subtle AD-related differences in WM connectivity than standard resolution DWI acquisitions.

## 1 Introduction

Decline in the microstructural composition of white matter (WM) over the course of adulthood contributes to a disconnection of distributed brain regions, and ultimately to a decline in cognition, even in healthy adults (Bennett & Madden, 2014; Salat, 2011). This decline in WM connectivity is magnified in adults with Alzheimer’s disease (AD; Chen et al., 2023; Gold et al., 2012; Mayo et al., 2017), and can be assessed *in vivo* using a noninvasive MRI technique termed diffusion-weighted imaging (DWI). Studies capitalizing on standard DWI acquisitions have been fundamental for furthering our understanding of WM disconnection in numerous diseases and conditions, such as traumatic brain injury, multiple sclerosis, and anxiety (Armstrong et al., 2024; Glenn et al., 2022; Lakhani et al., 2020). However, the relatively low spatial resolution of standard DWI acquisitions (voxel size ≥1.5 mm^3^) has limited the ability of prior studies to adequately resolve WM connectivity in regions with crossing fiber populations or high curvature. This issue becomes even more exacerbated in neurodegenerative disease and illustrates the need for more advanced DWI approaches, such as higher spatial resolution data acquisitions.

Multi-shot multiplexed sensitivity encoding (MUSE) DWI is a clinically feasible acquisition that achieves spatial resolutions ≤1 mm^3^ via simultaneous multi-slice imaging (Bruce et al., 2017; Chen et al., 2013; Ma et al., 2023). Importantly, MUSE DWI can be acquired on conventional, widely accessible 3T scanners, rather than specialized 7T scanners as in other high-resolution protocols (e.g., Vu et al., 2015), and can be obtained in a relatively short scan time of ~15 minutes. Studies of rodents (e.g., Crater et al., 2022) and younger adults (e.g., Chang et al., 2015) already suggest that higher spatial resolution DWI provides additional information regarding WM connectivity, when directly compared to measurements obtained from standard DWI. Our prior work further demonstrated that measures of WM connectivity from this specific MUSE acquisition were differentially related to chronological age than standard resolution measures, and that only the high-resolution measures were significant mediators of age-related decline in general fluid cognitive abilities (Merenstein et al., 2023). However, this earlier work has not yet been translated to AD, where the higher spatial resolution may better detect the group difference in structural connectivity due to AD-related increases in partial volume effects (Berlot et al., 2014; Jeon et al., 2012; Metzler-Baddeley et al., 2012; Vu et al., 2015).

Graph theoretical analyses are especially well-suited to assess AD-related differences in WM connectivity, because the brain comprised distinct networks of individual gray matter (GM) regions, which are modeled as nodes, as well as the WM connections between these nodes, which are modeled as edges (Bullmore & Sporns, 2009; Rubinov & Sporns, 2010; Wig, 2017). Previous applications of graph theory to standard DWI data have identified AD-related decreases in the efficiency of information transfer, primarily in the frontal lobe and default mode network (Dai et al., 2019; Lo et al., 2010). In addition to measures of network efficiency, graph theoretical analyses can also measure structural system segregation, where higher segregation is indicative of more separated (less integrated) networks. Healthy aging has been associated with increased segregation of WM networks, due to greater age-related declines in long-range WM connections than in short-range connections (Madden et al., 2024; Zhao et al., 2015). When applying this approach beyond healthy aging, using standard DWI data, participants who were at higher risk of developing AD-related dementia (based on biomarkers of amyloid beta [Aβ] and neurodegeneration) exhibited higher global network segregation than those at lower risk, and these differences persisted even after a 2-year follow-up (Pereira et al., 2018). Across the lifespan, graph theoretical metrics of structural connectivity are also lower in adults with an increased risk of developing AD due to genetic factors (i.e., apolipoprotein E ε4 carriers) relative to those who have a lower genetic risk (Brown et al., 2011; Ma et al., 2017; Mirza-Davies et al., 2022). However, a limitation of these prior studies examining AD-related differences in WM connectivity is that their standard DWI data consisted of larger voxel sizes (≥1.8 mm in-plane resolution), which is less ideal for constructing tractography-based measures of the structural connectome.

More fine-grained assessments of WM deterioration are critical as this deterioration is thought to occur prior to the accumulation of Aβ and possibly also AD-related tau pathology (Falangola et al., 2020; Hu et al., 2025; Wang et al., 2022) and may therefore serve as a valuable biomarker of adults at risk for later developing AD. This study will test whether high-resolution MUSE DWI (1 mm^3^ voxels; 1 µl volume) measures of structural connectivity predict AD better than standard DWI (1.5 mm^3^; 3.375 µl), as would be expected by the three-fold increase in spatial resolution. A sample of 24 participants with AD and 24 demographically matched controls completed an MRI scanning session where data from both DWI acquisitions was collected from all participants. We obtained graph theoretical measures of within- and between-network connectivity, and system segregation, from functionally-defined brain networks (Yeo et al., 2011).

For both acquisitions, we expected to replicate lower within-network and between-network connectivity, and increased system segregation, in participants with AD relative to healthy controls, in line with previous work in adults at risk of AD (Pereira et al., 2018). If the high-resolution acquisition improves the detection of AD-related differences in structural connectivity, then the high-resolution measures should reveal larger group differences and greater sensitivity and specificity to AD than the corresponding standard resolution measures, especially in frontal and default mode networks (Dai et al., 2019; Lo et al., 2010).

## 2 Methods

### 2.1 Participants

This study was conducted in compliance with the Code of Ethics of the World Medical Association (Declaration of Helsinki) for experiments involving humans and with the Institutional Review Board for Duke University Medical Center. All participants (and their caregivers, if necessary) provided written informed consent, were free of MRI contraindications (i.e., ferrous metal devices and implants), and were compensated for their time.

All participants completed a 1-hour. MRI scan, the Montreal Cognitive Assessment (MoCA; Nasreddine et al., 2005), and a brief demographic questionnaire. Twenty-four participants diagnosed with AD were recruited from the Duke University-University of North Carolina (UNC) at Chapel Hill Alzheimer’s Disease Research Center (Duke/UNC ADRC). Participants were diagnosed with AD based on a multidisciplinary clinical consensus panel comprised of neurologists, psychiatrists, neuropsychologists, and research coordinators (Jack et al., 2018). The panel takes into account patient and caregiver report of cognitive symptoms and functioning from the Clinical Dementia Rating (Morris, 1993), Functional Activities Questionnaire (Pfeffer et al., 1982), and Neuropsychiatric Inventory (Cummings et al., 1994); neuropsychological testing measures from the Uniform Data Set 3 (Besser et al., 2018); and the degree of brain atrophy assessed by T1-weighted anatomical MRIs. This study included all ADRC participants who were diagnosed with AD and had MRI data available at the time of data analyses in Fall 2024. At present, Aβ status is available for 16 of the 24 participants with AD using cerebrospinal fluid (CSF) measures (*n* = 15) from a Fujirebio immunoassay (Fujirebio, Ghent, Belgium) or positron emission tomography (*n* = 1) and confirmed that 14 out of 16 (87.5%) participants with AD who had these measures available exhibited Aβ positivity.

In addition, 24 control participants were selected from larger samples based on demographic information to match the patients, including 10 cognitively healthy controls recruited from the Duke/UNC ADRC and 14 controls recruited from an in-house dataset of high-functioning, community-dwelling volunteers. Normal cognition in the control participants recruited from the ADRC was determined using the same criteria previously described for the participants diagnosed with AD. All control participants recruited from the ADRC were negative for Aβ in their CSF measures. Formal clinical assessments or measures of Aβ were not obtained from the community-dwelling volunteers, but they were required to have intact general cognition (score ≥26) on the MoCA (Nasreddine et al., 2005), a score >50th percentile on the Wechsler Adult Intelligence Scale-III vocabulary subtest (Wechsler, 1997), a score < 15 on the Beck Depression Inventory (Beck, 1978), and normal (corrected) visual acuity (Snellen score ≤20/40) on the Freiburg Visual Acuity Test (Bach, 1996). The volunteers were also free of major neurological (e.g., epilepsy, stroke, psychosis) and medical (e.g., emphysema, uncontrolled hypertension) conditions, and were not taking any medications known to affect cognition (e.g., antidepressants, stimulants). Sample characteristics for each group are provided in Table 1.

**Table 1.** Participant characteristics.

Variable	Alzheimer’s disease	Controls	*t*/*χ*^2^	*p*-value
*N*	24	24		
Age				
Mean (SD)	69.08 (7.34)	68.95 (7.44)	0.000	0.999
Median (range)	69.50 (54-80)	70.00 (54-82)		
Sex				
Female (%)	16 (66.0%)	16 (66.0%)	0.000	1.000
Education (years)				
Mean (SD)	16.30 (3.10)	17.04 (1.47)	1.048	0.304
Median (range)	16.00 (12–20)	16.00 (14–20)		
Not reported	1 (4.5%)	0 (0.0%)		
Montreal Cognitive Assessment (MoCA)				
Mean (SD)	17.46 (5.59)	27.92 (2.12)	**8.974**	**<0.001**
Median (range)	19.00 (4–25)	28.00 (26–30)		
Race				
White (%)	18 (75.00%)	21 (87.50%)	0.710	0.400
Black (%)	5 (20.83%)	3 (12.50%)		
Asian (%)	1 (4.17%)	0 (0.0%)		
APOE				
e4+ (%)	14 (58.33%)	1 (4.17%	**7.942**	**0.005**
e4- (%)	8 (33.33%)	9 (37.50%		
Missing (%)	2 (8.33%	14 (58.33%)		

*Note*. Continuous variables are presented with their corresponding mean (standard deviation, SD) and median (range), with group differences assessed using two-sample sample *t*-tests. Categorical variables are presented as *n* (%), with group differences assessed using chi-square (*χ*^2^) tests. Significant group differences (*p*_uncorrected_ < 0.05; bolded) were only observed for the score on the Montreal Cognitive Assessment and Apolipoprotein E (APOE) genotype.

### 2.2 Imaging data acquisition

All imaging data were acquired at the Brain Imaging and Analysis Center at Duke University Medical Center from 2021 to 2024. Nearly half of the data (*n* = 15 AD, *n* = 10 controls) were acquired on a 3T GE MR750 MRI scanner equipped with an eight-channel head coil. In 2022, scanner upgrades were performed, and the remainder of the data (*n* = 9 AD, *n* = 14 controls) were acquired on a 3T GE Ultra High-Performance MRI scanner equipped with a 48-channel head coil. Participants wore earplugs to reduce scanner noise, and foam pads were used to minimize head motion.

For all participants, a high-resolution T1-weighted image was acquired using a 3D fast inverse-recovery-prepared spoiled gradient recalled (SPGR) sequence with the following parameters: repetition time (TR) = 2,232.8 ms, echo time (TE) = 3.2 ms, inversion recovery time = 900 ms, flip angle = 8°, voxel size = 1 x 1 x 1 mm, acquisition matrix = 256 x 256 x 124 mm, and sensitivity encoding (SENSE) factor = 2.

For all participants, whole-brain standard resolution DWI data were acquired using a single-shot spin-echo echo planar imaging (EPI) sequence with the following parameters: TR = 4,620 ms, TE = 64.1 ms, flip angle = 90°, voxel size = 1.5 x 1.5 x 1.5 mm, acquisition matrix = 144 x 144 x 83 mm, and multiband factor = 3. Diffusion-weighted gradients were applied in 90 directions with *b* values of 1,500 and 3,000 s/mm^2^ and with two non-diffusion-weighted *b* = 0 images. For all participants, except for one control participant, a second diffusion sequence was acquired with six phase-encoding directions of opposite polarity using identical parameters, except that TR = 4,971 ms.

For all participants, a high-resolution MUSE DWI acquisition was also acquired. For 19 participants (*n* = 7 AD, *n* = 12 controls), whole-brain high-resolution DWI data were acquired using a four-shot MUSE spin-echo EPI sequence with the following parameters: TR = 13,000 ms, TE = 58 ms, flip angle = 90°, voxel size = 1 x 1 x 1 mm, acquisition matrix = 256 x 256 x 116 mm, and multiband factor = 1. Diffusion-weighted gradients were applied in 15 directions with a *b* value of 800 s/mm^2^ and with two non-diffusion-weighted *b* = 0 images. For the remaining 29 participants (*n* = 17 AD, *n* = 12 controls), whole-brain high-resolution DWI data were acquired using a four-shot MUSE spin-echo EPI sequence with the following parameters: TR = 8,767 ms, TE = 60.8 ms, flip angle = 90°, voxel size = 1 x 1 x 1 mm, acquisition matrix = 164 x 328 x 122 mm, and multiband factor = 2. Diffusion-weighted gradients were applied in 25 directions with a *b* value of 800s/mm^2^ and with two non-diffusion-weighted *b* = 0 images.

During scanning, we also acquired data from resting-state functional MRI, susceptibility-weighted angiography, and fluid attenuated inversion recovery imaging, which will be reported in separate articles (Merenstein et al., 2024).

### 2.3 DWI data processing

We processed the raw DWI data using MRtrix3 (Tournier et al., 2019) and FSL (FMRIB’s Software Library; Smith et al., 2004). The data were first denoised (*dwidenoise*) and then corrected for motion and eddy current-induced distortions (*dwifslpreproc*). The standard resolution data were additionally corrected for susceptibility-induced off-resonance distortions (*topup*), except for one control participant who did not have reverse polarity data available. Lastly, all data were bias-corrected (*dwibiascorrect*) and non-brain tissue was removed to generate a whole-brain mask (*dwi2mask*). All processed DWI scans were visually inspected by a trained researcher and found acceptable for the degree of brain mask coverage, quality of corrections for motion and susceptibility-induced distortions, and the absence of MR artifacts.

### 2.4 Structural connectivity estimation

To assess structural connectivity, we derived fiber orientation distribution (FOD) maps using multi-shell, multi-tissue constrained spherical deconvolution (Jeurissen et al., 2014; Tournier et al., 2004, 2019). This process was conducted individually for each participant and each DWI acquisition and was based on the *dhollander* tissue-specific response function. This response was derived from three compartments (GM, WM, CSF) in the multi-shell standard resolution data or two compartments (WM, CSF) in the single-shell high-resolution data (Dhollander et al., 2019). Next, a GM/WM boundary mask was generated from the high-resolution T1-weighted image using the *5ttgen* command (Smith et al., 2012; Tournier et al., 2019). For each DWI acquisition, we used FSL’s *flirt* to register the average skull-stripped, preprocessed mean *b* = 0 image to the GM/WM boundary mask using a linear registration with six degrees of freedom (Jenkinson & Smith, 2001; Jenkinson et al., 2002; Smith et al., 2004) and applied the inverse of this transformation matrix to register the GM/WM boundary mask to native diffusion space (*transformconvert*, *mrtransform*). Using the FOD maps, we performed anatomically constrained probabilistic tractography, in native space, using a standard pipeline from MRtrix, where streamlines are both seeded from and terminated within the GM/WM boundary mask (Smith et al., 2012). We set the minimum streamline length to 1 mm, maximum length to 250 mm, and FOD cutoff to 0.06. For each participant and DWI acquisition, we generated 10 million tracts (*tckgen*) and used spherical-deconvolution informed filtering of tractograms 2 (*tcksift2*) in MRtrix to assign a weight to each streamline, relative to the underlying apparent fiber density (Smith et al., 2015). Lastly, for each participant, the cells in the matrix were multiplied by a proportionality coefficient parameter, *μ*, which allows for inter-subject comparisons even when there are group differences in the total number of reconstructed streamlines or pathological decreases in fiber density (Smith et al., 2020).

For each participant and DWI acquisition, we transformed the streamlines from native diffusion space to a standard MNI 152 1 x 1 x 1 mm template (*tcktransform*). Symmetrical structural connectivity matrices were created using *tck2connectome* to assign the 10 million weighted streamlines to the 246 cortical and subcortical nodes from the Brainnetome Atlas (Fan et al., 2016). The endpoint of each streamline was assigned to the nearest GM node using a 2 mm radial search (Smith et al., 2015). In the resulting 246 x 246 matrices, each cell represents the sum of the weighted contribution of the streamlines connecting that pair of nodes, scaled to account for differences in node volume (Hagmann et al., 2008; Tournier et al., 2019). As recommended by Civier et al. (2019), we did not remove cells with few or no connections assigned to them.

**Fig. 1.**
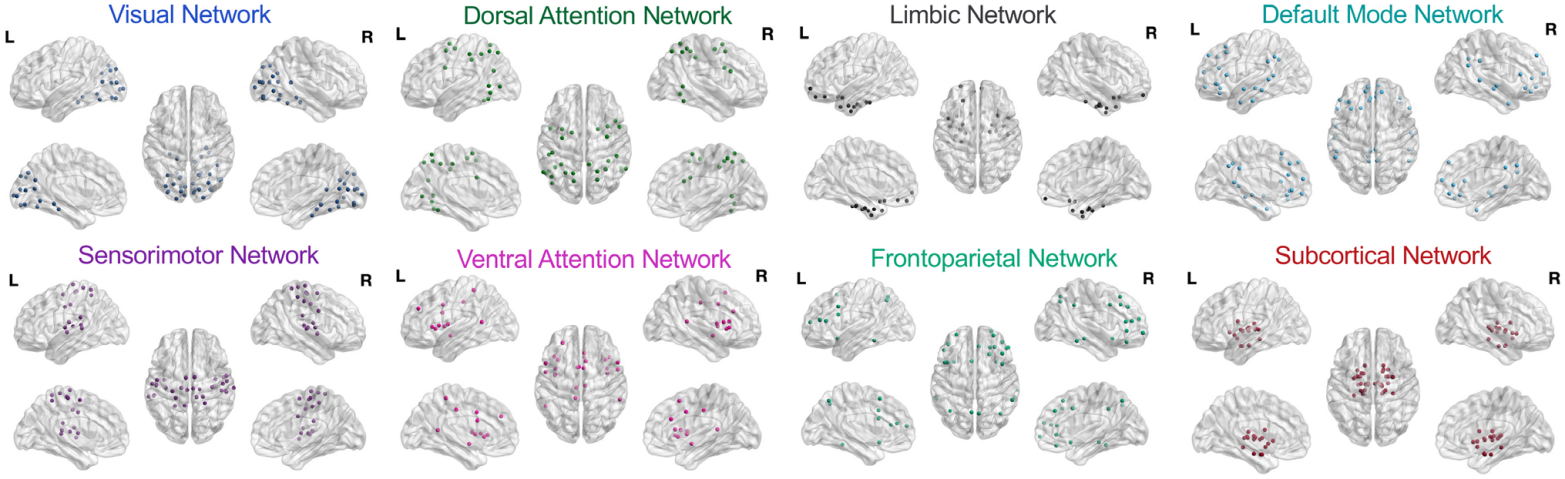
Network partition. The assignment of the 210 cortical and 36 subcortical nodes from the Brainnetome atlas (Fan et al., 2016) to their corresponding functionally-defined brain networks are displayed separately for each of the eight networks of interest, including the seven cortical networks from the Yeo et al. (2011) partition and an eighth subcortical network.

In the final step, we used the Brain Connectivity Toolbox (Rubinov & Sporns, 2010) to estimate three graph theoretical measures of connectivity strength: within-network connectivity, between-network connectivity, and system segregation (i.e., the ratio of within-network connectivity to between-network connectivity, divided by within-network connectivity). System segregation reflects the degree to which the functional networks are distinct from each other, with higher values being indicative of more distinct networks. Each of the 210 cortical nodes were assigned to either the dorsal attention, default mode, frontoparietal, limbic, sensorimotor, ventral attention, or visual networks (Yeo et al., 2011), and the 36 subcortical nodes were combined into their own network (Fig. 1), similar to prior work (Capogna et al., 2022; Luo et al., 2020). Each measure of connectivity strength was obtained at the global, whole-brain level as well as separately for each of the eight individual networks.

### 2.5 Statistical analysis

All statistical analyses were performed using a combination of RStudio (4.3.1; http://www.rstudio.com), MATLAB (2023a; https://www.mathworks.com), and JASP (0.17.1; https://jasp-stats.org). A *χ*^2^ test indicated that distribution of the different scanner features did not significantly differ between the two groups, *χ*^2^ = 2.087, *p*_uncorrected_ = 0.149. Nonetheless, to better account for potential impacts of this change, we harmonized the structural connectivity data using the *neuroHarmonize* Python tool version 2.4.5 (Pomponio et al., 2020). The two different scanner configurations were treated as the site variable, and age and group were included as covariates. Relations between the harmonized structural connectivity measures from each acquisition were assessed using Pearson correlations.

To assess group differences in structural connectivity, we used repeated measures ANOVA with group (AD, controls) as a between-subjects variable and acquisition (high-resolution, standard) as a within-subjects variable, separately for each combination of connectivity and network. We followed up on significant interactions using post hoc *t*-tests with the Holm-Bonferroni method applied for multiple comparison correction. The protocol for acquiring biomarker data was not yet set up for eight participants with AD who belong to the earliest subset of the sample recruited by the Duke-UNC ADRC. To ensure that structural connectivity did not differ by A*β* pathology status before further analysis, we conducted unpaired *t*-tests between the 14 participants with AD confirmed as A*β-*positive and the eight participants with AD with missing A*β* data. Results confirmed that there were no significant subgroup differences for either the standard or high-resolution graph theoretical measures at either the global or network level, *p_uncorrected_* ≥ 0.055.

Next, to test our primary hypothesis that high-resolution DWI improves the characterization of WM disconnection in AD, we performed receiver operating characteristic (ROC) analyses to assess whether the high-resolution DWI protocol was more sensitive or specific to differences in structural connectivity between healthy controls and participants with AD. We first obtained the actual observed values by performing binomial logistic regression models with structural connectivity predicting group, separately for each DWI acquisition. We then used the *predict* function from the *stats* package in R to obtain the predicted values from the fitted regression model, with values being assigned a 0 or 1 depending on whether they were below or above a standard threshold of 0.5 (i.e., assuming equal sensitivity and specificity). The actual observed values and predicted values were combined into a confusion matrix and then used as input for the *caret* package to calculate sensitivity and specificity. Lastly, we calculated the probabilities of each data point being classified as the correct group and plotted these probabilities against their true observed value using the *roc* function in the *pROC* package. Finally, we derived area under the curve (AUC) values using the *auc* function, where AUC values between 0.7 to 0.8 were considered to be acceptable and 0.8 to 0.9 were considered to be excellent (Mandrekar, 2010). Significant differences between ROC curves were tested using the *roc.test* function, which compares correlated ROC curves based on a bootstrapping procedure with 2,000 replacements.

## 3 Results

### 3.1 Correlations between acquisitions

First, we used separate Pearson correlations to examine associations between the high-resolution and standard resolution structural connectivity measures

#### 3.1.1 Within-network connectivity

The global measure was highly correlated between acquisitions, *r* = 0.598, *p* < 0.001, with the network level correlations ranging from *r* = 0.435, *p* = 0.002, in the ventral attention network to *r* = 0.699, *p* < 0.001 in the dorsal attention network (Table 2). Measures of within-network connectivity from the subcortical network were not significantly correlated, *r* = 0.183, *p* = 0.214.

**Table 2.** Correlations between acquisitions.

	*r* standard-high-resolution		
	Within-network	Between-network	System segregation
AVG	0.598***	0.579***	0.733***
VIS	0.644***	0.554***	0.614***
SMN	0.679***	0.492***	0.479***
DAN	0.699***	0.624***	0.468***
VAN	0.435**	0.489***	0.333*
LMB	0.674***	0.700***	0.631***
FPN	0.628***	0.589***	0.709***
DMN	0.451**	0.563***	0.511***
SUB	0.183	0.768***	0.762***

*Note.* Networks are abbreviated as dorsal attention (DAN), default mode (DMN), frontoparietal (FPN), sensorimotor (SMN), subcortical (SUB), or ventral attention (VAN) or the global measure that was averaged across all networks (AVG). Bolded text indicates networks that were significantly correlated between acquisitions. ***p < 0.001, **p < 0.01, **p < 0.05.

#### 3.1.2 Between-network connectivity

The global measure was highly correlated between acquisitions, *r* = 0.579, *p* < 0.001, with the network level correlations ranging from *r* = 0.489, *p* < 0.001, in the ventral attention network to *r* = 0.768, *p* < 0.001 in the subcortical network (Table 2).

#### 3.1.3 System segregation

The global measure was highly correlated between acquisitions, *r* = 0.733, *p* < 0.001, with the network level correlations ranging from *r* = 0.468, *p* < 0.001, in the dorsal attention network to *r* = 0.762, *p* < 0.001 in the subcortical network (Table 2).

### 3.2 Group differences in global connectivity measures

To assess whether WM structural connectivity differed in participants with AD, and whether these estimates varied by acquisition, we conducted repeated measures Group x Acquisition ANOVAs, separately for each global measure of connectivity (within-network, between-network, or system segregation). Significant main effects and interactions were probed using post hoc *t*-tests with the Holm-Bonferroni method applied for multiple comparison correction. Values are presented as the mean difference ± standard error.

#### 3.2.1 Within-network connectivity

**Fig. 2.**
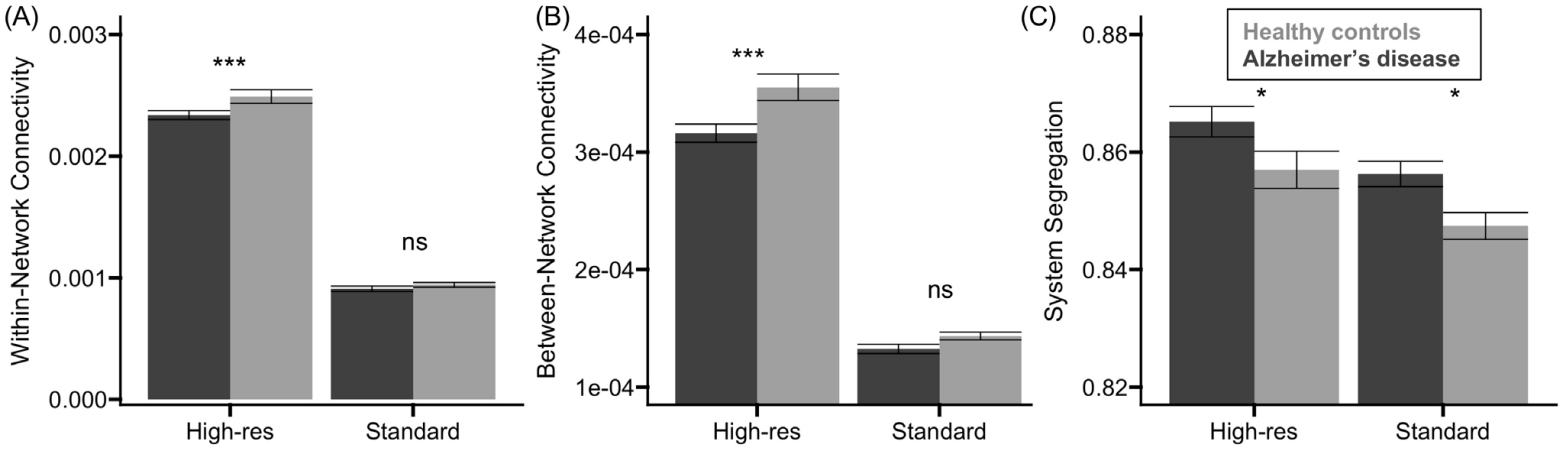
Group differences in global structural connectivity. Relative to healthy controls (light gray), analyses of variance (ANOVA) indicated that participants with AD (dark gray) exhibited significantly lower (A) within-network connectivity and (B) between-network connectivity for only the high-resolution acquisition. (C) System segregation was measured as the difference between mean within-network connectivity and mean between-network connectivity, divided by mean within-network connectivity, and was significantly higher in participants with AD relative to healthy controls for both acquisitions. Error bars represent the standard error of the mean. ns = non-significant difference. Asterisks * denote significant group differences.

There was a significant main effect of acquisition, *F*(1, 46) = 2971.977, *p* < 0.001, *η²_p_* = 0.985 (Fig. 2A), indicating that connectivity was higher for the high-resolution than the standard acquisition (mean difference = 0.001 ± 2.730e5), and group, *F*(1, 46) = 4.473, *p* = 0.034, *η²_p_* = 0.089 (Fig. 2A), where connectivity was lower for participants with AD relative to controls (mean difference = 9.208e5 ± 4.354e5). A significant Group x Acquisition interaction, *F*(1, 46) = 4.767, *p* = 0.034, *η²_p_* = 0.094, indicated that the group difference in connectivity was significant for the high-resolution acquisition, *t*(46) = 2.952, *p_holm_* = 0.008, with lower connectivity for participants with AD relative to controls. The group difference was not significant for the standard resolution acquisition, *p_holm_* = 0.529 (Fig. 2A).

#### 3.2.2 Between-network connectivity

There was a significant main effect of acquisition, *F*(1, 46) = 1116.319, *p* < 0.001, *η²_p_* = 0.960 (Fig. 2B), indicating that connectivity was higher for the high-resolution than the standard acquisition (mean difference = 1.982e4 ± 5.931e6), and group, *F*(1, 46) = 9.206, *p* = 0.004, *η²_p_* = 0.167 (Fig. 2B), where connectivity was lower for participants with AD relative to controls (mean difference = 2.578e5 ± 8.497e6). A significant Group x Acquisition interaction, *F*(1, 46) = 4.998, *p* = 0.030, *η²_p_* = 0.098, indicated that the group difference in connectivity was significant for the high-resolution acquisition, *t*(46) = 3.768, *p_holm_* < 0.001, with lower connectivity for participants with AD relative to controls. Similar to the pattern observed for within-network connectivity, the group difference was not significant for the standard resolution acquisition, *p_holm_* = 0.230 (Fig. 2B).

#### 3.2.3 System segregation

There was a significant main effect of acquisition, *F*(1, 46) = 40.063, *p* < 0.001, *η²_p_* = 0.466 (Fig. 2C), indicating that the estimated degree of network segregation was higher for the high-resolution than for the standard acquisition (mean difference = 0.009 ± 0.001), and group, *F*(1, 46) = 6.497, *p* = 0.014, *η²_p_* = 0.124 (Fig. 2C), where segregation was higher for participants with AD relative to controls (mean difference = 0.009 ± 0.003). The Group x Acquisition interaction was not significant, *p* = 0.821 (Fig. 2C).

### 3.3 Group differences in local connectivity measures

Next, we used the exact same approach as the global measures to assess differences in local measures of connectivity strength between healthy controls and participants with AD, with separate ANOVAs for each combination of network and measure of connectivity.

#### 3.3.1 Within-network connectivity

**Fig. 3.**
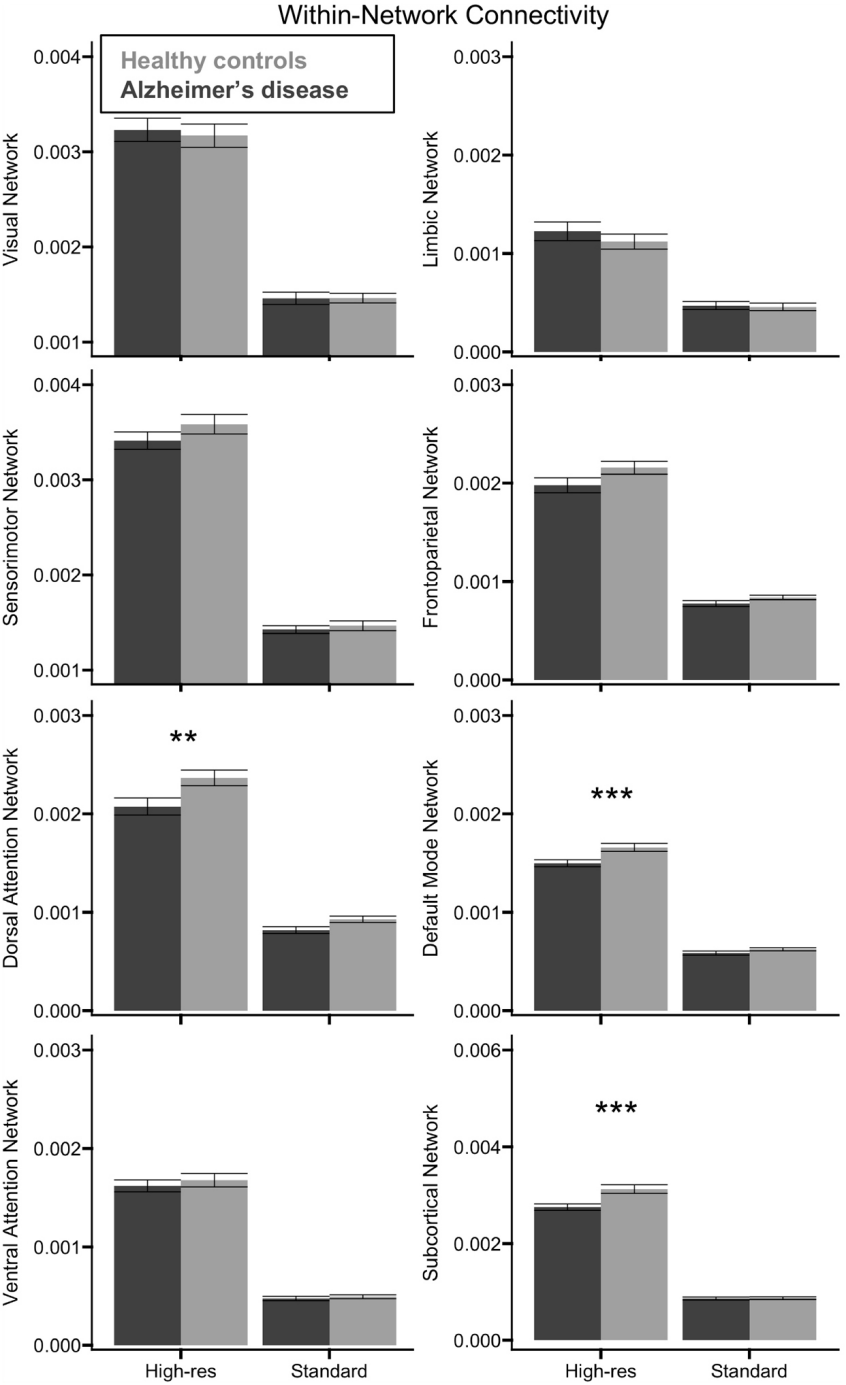
Group differences in local within-network connectivity. Relative to healthy controls (light gray), participants with AD (dark gray) exhibited significantly lower within-network connectivity in the dorsal attention and frontoparietal networks across acquisitions, but the decrease in connectivity for the default mode and subcortical networks was only significant when assessed by the high-resolution (high-res) acquisition. Error bars represent the standard error of the mean. *p_holm_ < 0.05, **p_holm_ < 0.01, ***p_holm_ < 0.001.

There was a significant main effect of acquisition on all eight networks, *F*(1, 46) ≥ 225.794, *p* < 0.001, *η²_p_* ≥ 0.831 (Fig. 3), with consistently higher connectivity for the high-resolution than the standard acquisition. There was also a significant main effect of group for the dorsal attention, default mode, and subcortical networks, *F*(1, 46) ≥ 6.886, *p* ≤ 0.012, *η²_p_* ≥ 0.130 (Fig. 3), with lower connectivity in these networks for participants with AD relative to controls. Significant Group x Acquisition interactions were observed for the default mode and subcortical networks, *F*(1, 46) ≥ 6.275, *p* ≤ 0.016, *η²_p_* ≥ 0.120. In these two networks, the group difference in connectivity was significant for the high-resolution acquisition, *t*(47) ≥ 3.828, *p_holm_* ≤ 0.001, with lower connectivity for participants with AD than the controls in each case. However, these group differences were not significant for the standard resolution acquisition, *p_holm_* ≥ 0.369 (Fig. 3).

#### 3.3.2 Between-network connectivity

**Fig. 4.**
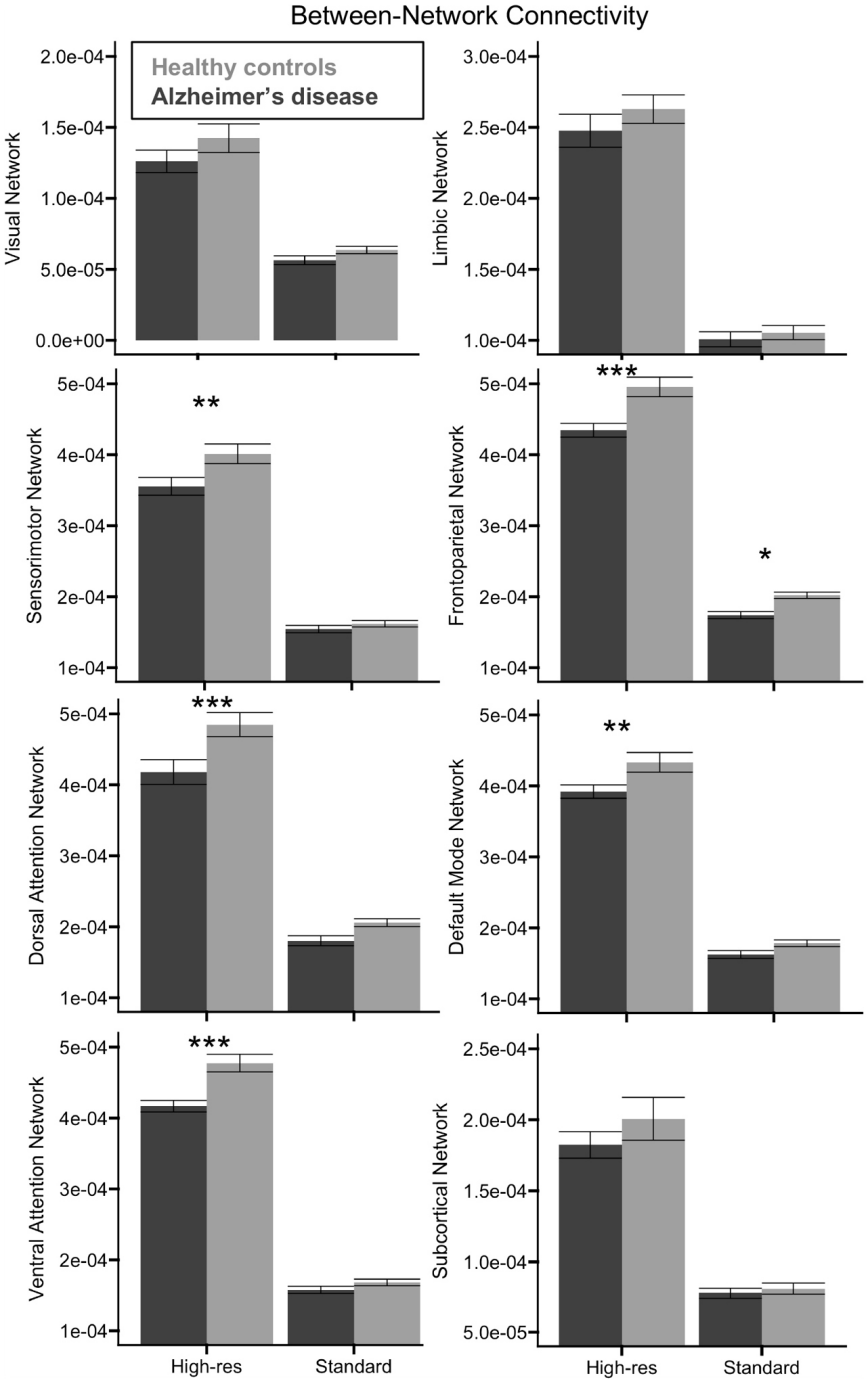
Group differences in local between-network connectivity. Relative to healthy controls (light gray), participants with AD (dark gray) exhibited significantly lower between-network connectivity in the visual and frontoparietal networks across acquisitions, but the decrease in connectivity for the sensorimotor, dorsal attention, ventral attention, default mode, and subcortical networks was only significant when assessed by the high-resolution (high-res) acquisition. Error bars represent the standard error of the mean. *p_holm_ < 0.05, **p_holm_ < 0.01, ***p_holm_ < 0.001.

There was a significant main effect of acquisition on all eight networks, *F*(1, 46) ≥ 176.102, *p* < 0.001, *η²_p_* ≥ 0.763 (Fig. 4), with consistently higher connectivity for the high-resolution than the standard acquisition. There was also a significant main effect of group for the sensorimotor, dorsal attention, ventral attention, frontoparietal, and default mode networks, *F*(1, 46) ≥ 5.494, *p* ≤ 0.023, *η²_p_* ≥ 0.107 (Fig 4), where connectivity in these networks was lower for participants with AD relative to controls. Significant Group x Acquisition interactions were observed for the sensorimotor, ventral attention, and frontoparietal networks, *F*(1, 46) ≥ 4.787, *p* ≤ 0.034, *η²_p_* ≥ 0.094. In the frontoparietal network, this interaction indicated that the group difference in connectivity was significantly larger for the high-resolution, *t*(46) = 4.746, *p_holm_* < 0.001, relative to standard resolution, *t*(47) = 2.180, *p_holm_* = 0.032, acquisition. In the sensorimotor and ventral attention networks, the group difference was significant for the high-resolution acquisition, *t*(46) ≥ 3.253, *p* ≤ 0.003, but not the standard resolution acquisition, *p_holm_* ≥ 0.370 (Fig. 4).

#### 3.3.3 System segregation

There was a significant main effect of acquisition on the dorsal attention, ventral attention, default mode, and subcortical networks, *F*(1, 46) ≥ 14.306, *p* < 0.001, *η²_p_* ≥ 0.237, indicating that segregation was higher for the high-resolution than the standard acquisition. There was a significant main effect of group for the visual network, *F*(1, 46) = 6.431, *p* = 0.015, *η²_p_* = 0.123, where segregation was higher for participants with AD relative to controls. There were no significant Group x Acquisition interactions for any network, *p* ≥ 0.201.

### 3.4 Sensitivity and specificity analyses

Next, to assess whether the high-resolution acquisition was more sensitive or specific to AD-related differences in structural connectivity than the standard resolution acquisition, we performed ROC analyses on the global or local measures of connectivity that exhibited a significant group difference for at least one acquisition (Figs. 3 and 4), and tested for significant differences in the correlated ROC curves between acquisitions using a bootstrapping method.

#### 3.4.1 Within-network connectivity

**Fig. 5.**
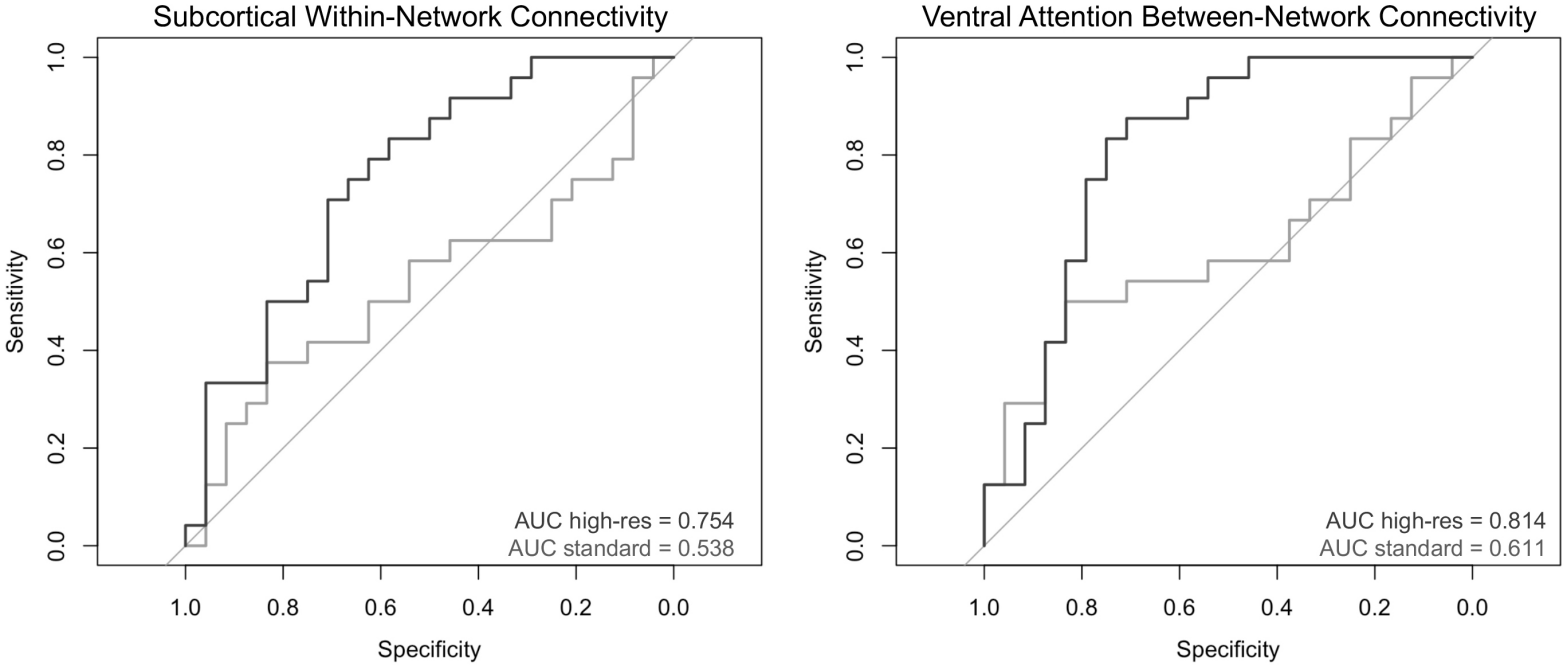
Receiver operating characteristic (ROC) plots. ROC plots illustrate the area under the curve (AUC) for subcortical within-network connectivity and ventral attention between network-connectivity, separately for the high-resolution (high-res; dark gray) or standard resolution (light gray) acquisition. A higher AUC value and a curve further away from the diagonal is indicative of higher sensitivity and specificity. Both AUC curves were significantly higher for the high-resolution relative to standard resolution acquisition.

Acceptable AUC values were found for the default mode (0.741) and subcortical (0.754) networks for the high-resolution acquisition (Table 3). Relative to the standard acquisition, the high-resolution acquisition exhibited larger AUC values for the global measure and all three networks. The difference in ROC curves between acquisitions was significant for the subcortical network, *p* = 0.026 (Fig. 5), but not for the global measure or the dorsal attention and default mode networks, *p* ≥ 0.124 (Table 3).

**Table 3.** Sensitivity and specificity analyses.

	Standard resolution			High-resolution		
	Sensitivity	Specificity	AUC	Sensitivity	Specificity	AUC
*Within-Network Connectivity*						
AVG	0.667	0.542	0.597	0.667	0.750	0.689
DAN	0.625	0.542	0.667	0.667	0.625	0.679
DMN	0.667	0.583	0.613	0.667	0.708	0.741
SUB*	**0.458**	**0.583**	**0.538**	**0.625**	**0.750**	**0.754**
*Between-Network Connectivity*						
AVG	0.708	0.625	0.681	0.708	0.750	0.773
SMN	0.583	0.625	0.592	0.667	0.708	0.719
DAN	0.708	0.583	0.707	0.667	0.708	0.743
VAN*	**0.458**	**0.583**	**0.611**	**0.750**	**0.792**	**0.814**
FPN	0.708	0.625	0.800	0.708	0.708	0.793
DMN	0.667	0.583	0.672	0.708	0.625	0.707
*System segregation*						
AVG	0.542	0.625	0.694	0.583	0.625	0.656
VIS	0.583	0.542	0.662	0.583	0.667	0.689

*Note.* Networks are abbreviated as dorsal attention (DAN), default mode (DMN), frontoparietal (FPN), sensorimotor (SMN), subcortical (SUB), or ventral attention (VAN) or the global measure that was averaged across all networks (AVG). AUC = area under the curve. Bolded text and networks marked with an asterisk had significantly higher AUCs for the high-resolution than standard resolution acquisition.

#### 3.4.2 Between-network connectivity

Excellent AUC values were found in the ventral attention network (0.814) for the high-resolution acquisition, and in the frontoparietal network (0.800) for the standard resolution acquisition (Table 3). Acceptable AUC values were found in the sensorimotor (0.719), dorsal attention (0.743), frontoparietal (0.793), and default mode (0.707) networks for the high-resolution acquisition, and in the dorsal attention (0.707) network for the standard resolution acquisition (Table 3). Relative to the standard resolution acquisition, the high-resolution acquisition exhibited larger AUC values in for the global measure and all networks except for the frontoparietal network (Table 3). The difference in ROC curves between acquisitions was significant for the ventral attention network, *p* = 0.022 (Fig. 5), but not for the global measure or the other four networks, *p* ≥ 0.097 (Table 3).

#### 3.4.3 System segregation

Acceptable AUC values were not observed for the global measure for the high-resolution (0.656) or standard resolution (0.694) acquisition, or for the visual network for the high-resolution (0.689) or standard resolution (0.662) acquisition (Table 3). Relative to the standard resolution acquisition, the high-resolution acquisition exhibited a larger AUC value for the visual network but a smaller AUC value for the global measure (Table 3), but the difference in ROC curves between acquisitions was not significant for either measure, *p* ≥ 0.513 (Table 3).

### 3.5 Structural connectivity and MoCA scores

Lastly, we tested whether the increased sensitivity and specificity of the high-resolution acquisition to group differences in structural connectivity resulted in an increased ability to predict general cognitive status, as assessed by scores on the MoCA. We conducted separate Pearson correlations between each global structural connectivity measure and MoCA performance, separately for each group (AD or healthy controls), with false discovery rate (FDR) procedures applied for multiple comparison correction (Benjamini & Hochberg, 1995). No significant correlations were identified for either group for either the high-resolution, *p*_FDR_ ≥ 0.526, or standard resolution, *p*_FDR_ ≥ 0.549, acquisition.

## 4 Discussion

Previous standard resolution DWI studies have identified AD-related increases in WM degradation relative to healthy controls, but these studies were limited in their ability to adequately resolve differences in voxels containing multiple fiber populations or small, tightly curved WM regions. To address this limitation, the current study acquired DWI data from both a standard resolution acquisition and a novel, high-resolution multi-shot MUSE acquisition within a sample of participants with AD and demographically matched healthy controls. Graph theoretical analyses, as expected, identified significantly higher structural system segregation in participants with AD relative to healthy controls for both DWI acquisitions, which was driven by larger AD-related decline in between-network relative to within-network connectivity. However, the high-resolution acquisition was more sensitive and specific to differences in both within- and between-network connectivity, especially in the ventral attention and subcortical networks. Together, these findings suggest that this clinically feasible, high-resolution DWI methodology may detect more subtle AD-related differences in WM connectivity than standard resolution DWI acquisitions.

Our first main finding is that we observed a pattern of increased structural system segregation in participants with AD relative to healthy controls, extending similar patterns found with graph theoretical analyses of DWI data for healthy adults (Madden et al., 2024; Zhao et al., 2015) and adults at risk of AD (Pereira et al., 2018). For both the high-resolution and standard acquisitions, the increase in system segregation was driven by larger AD-related decreases in between-network connectivity than within-network connectivity. Importantly, however, the high-resolution data identified significant AD-related decreases in both between- and within-network connectivity, whereas these decreases were not significant for the standard resolution measures. Finding that the high-resolution acquisition better detected AD-related differences in within-network connectivity is expected, as this measure should reflect the characteristics of streamlines for tightly curved u-fibers (Guevara et al., 2020). These streamlines would benefit from higher spatial resolution more than the long-range connections between networks, although the high-resolution acquisition similarly improved the detection of AD-related differences the degree of between-network connectivity as well. The loss of WM connectivity in AD likely reflects a combination of processes, including demyelination and damage to oligodendrocytes (Nasrabady et al., 2018), neuroinflammation (Garnier-Crussard et al., 2023), and cerebral amyloid angiopathy (Shirzadi et al., 2023). Regardless of the specific mechanisms, the current findings suggest that AD is characterized by more distinct (less integrated) structural networks when compared to healthy controls, whether assessed by standard or high-resolution DWI data. However, the estimation of AD-related increases in system segregation by standard acquisitions may be biased if these acquisitions are less sensitive to subtle AD-related loss of WM fibers within- and between individual networks when compared to higher spatial resolution DWI.

At the network level, AD-related decreases in between-network connectivity were evident for the frontoparietal network across acquisitions, and in the sensorimotor, dorsal attention, ventral attention, and default mode networks for the high-resolution acquisition, which is in line with predictions based on prior work observing similar AD-related effects in frontal and default mode regions (Dai et al., 2019; Lo et al., 2010; Mito et al., 2018). For within-network connectivity, AD-related decreases were observed in the dorsal attention and frontoparietal networks across acquisitions, and in the default mode and subcortical networks for the high-resolution acquisition. ROC analyses confirmed that the high-resolution measures of connectivity were more sensitive and specific (larger AUC values) to AD when compared to the corresponding standard resolution measures. Specifically, the high-resolution acquisition significantly increased the ability to predict AD from within-network connectivity of the subcortical network, and from between-network connectivity of the ventral attention network. These findings support the notion that high-resolution DWI may better estimate disease-related effects on the structural connectome, relative to standard resolution, extending previous work in rodents (Anderson et al., 2020; Crater et al., 2022), younger adults (Caiazzo et al., 2018; Chang et al., 2015), and healthy adults across the lifespan (Merenstein et al., 2023). The increased sensitivity of the MUSE acquisition was observed in spite of the higher angular resolution of the standard spatial resolution data (90 diffusion-weighted directions). Together, these findings suggest that, when deciding on specific DWI acquisition parameters, studies may wish to prioritize spatial resolution at the expense of angular resolution (Jones et al., 2020).

Results from this experiment will be informative for future studies that may wish to use this clinically feasible, high-resolution MRI methodology for studying WM disconnection in AD and its relation to other neural measures. For example, adults with AD exhibit positive correlations between structural and functional connectivity (the latter of which has typically been assessing using resting-state functional MRI), whereas healthy older adults exhibit negative correlations (Madden et al., 2020; Steward et al., 2023; Xu et al., 2022; Zhang et al., 2021). Based on the present finding that high-resolution DWI is more sensitive to AD-related differences in WM connectivity relative to standard resolution DWI, it follows that high-resolution DWI may also be more sensitive to aberrant structure-function relations. Another important potential future direction in this line of work is to use this high-resolution acquisition to assess relations between WM connectivity and AD-related Aβ and tau pathologies. For example, there is some evidence that WM degradation occurs before and independently of Aβ accumulation (Falangola et al., 2020; Hu et al., 2025), whereas relations between WM degradation and tau pathology are more mixed (Hu et al., 2025; Pereira et al., 2019; Wang et al., 2022). We did not observe significant associations between structural connectivity and MoCA scores here, despite our previous work demonstrating that this specific high-resolution MUSE acquisition better predicted age-related differences in tests of fluid, speed-dependent cognition across two separate studies of healthy adults across the lifespan (Merenstein et al., 2023, 2025). The current null finding may reflect the purpose of the MoCA as being a brief screening tool of general cognition with high sensitivity and specificity for detecting mild cognitive impairment (Nasreddine et al., 2005), and future investigations of structural connectivity and cognition may therefore benefit from test batteries targeting more specific domains of cognitive performance.

One limitation of the current study is that assessments of Aβ positivity were not obtained from 14 (out of 24) healthy controls, although these participants were characterized as high-functioning by a 2.5-hour. psychometric testing and health screening session completed prior to MRI scanning. In addition, Aβ positivity status has not currently been assessed for all 24 of the participants with AD, and for two of whom it was assessed, they were not deemed as being Aβ positive and may therefore have non-AD dementia. Future studies should aim for more homogenous case groups, with complete biomarker data, and also assess the value of MUSE DWI for identifying other common etiologies of dementia (e.g., frontotemporal dementia, Lewy body disease). Another limitation is the overall sample size of this study, which makes it difficult to reliably disentangle potential moderating effects of the two high-resolution acquisitions on structural connectivity. Future studies are needed to more comprehensively examine these potential differences, especially whether there is any increase in explanatory power achieved by increasing angular resolution (Caiazzo et al., 2018). Although a chi-square test suggested that the scanner upgrades did not differ between participants with AD and controls, we nonetheless chose to harmonize the current dataset to better account for possible impacts of this change on the observed patterns of structural connectivity.

## 5 Conclusions

In closing, the current DWI study demonstrated several differences between a high-resolution multi-shot MUSE acquisition and a standard resolution acquisition, in characterizing AD-related features of structural connectivity. Relative to demographically matched controls, participants with AD exhibited lower structural connectivity and increased system segregation, reflecting more distinct and less interconnected brain networks. Although this pattern of AD-related increases in structural system segregation was observed for both DWI acquisitions, the high-resolution acquisition was better at capturing declines in within- and between-network connectivity than the standard resolution acquisition, especially for networks known to be vulnerable to AD-related neurodegenerative processes (e.g., default mode network). Finding that these clinically feasible high-resolution measures are more sensitive to AD-related differences in the structural connectome suggests that this novel methodology may assist with the early identification of adults who are at risk for cognitive decline.

## Data Availability

The deidentified behavioral and preprocessed neuroimaging data from the 14 community-dwelling adults are available upon reasonable request to the corresponding author and a data sharing agreement will be required. The behavioral and neuroimaging data for any participant recruited from the Duke-UNC ADRC can be obtained by completing a resource request form on their website (https://dukeuncadrc.org). The code that was used for processing the imaging data and conducting data analyses has been uploaded to the GitHub profile of the corresponding author (https://github.com/j-merenstein/high-res_DWI_AD).
